# Lumbar degeneration and quality of life in patients with lumbar disc herniation: a case-control long-term follow-up study

**DOI:** 10.2340/17453674.2024.39944

**Published:** 2024-02-02

**Authors:** Sebastian PONTÉN, Tobias LAGERBÄCK, Sebastian BLOMÉ, Karin JENSEN, Mikael SKORPIL, Paul GERDHEM

**Affiliations:** 1 Department of Orthopaedics and Hand Surgery, Uppsala University Hospital, Uppsala; 2 Department of Clinical Science, Intervention and Technology (CLINTEC), Karolinska Institutet, Stockholm; 3 Department of Clinical Neuroscience, Division of Neuro, Karolinska Institutet, Stockholm; 4 Department of Molecular Medicine and Surgery, Karolinska Institutet, Stockholm; 5 Department of Neuroradiology, Karolinska University Hospital, Stockholm; 6 Department of Surgical Sciences, Uppsala University, Uppsala, Sweden

## Abstract

**Background and purpose:**

Adults treated surgically for lumbar disc herniation in adolescence have a higher degree of lumbar disc degeneration than controls. We aimed to establish whether the degree of lumbar degeneration differs at diagnosis or at follow-up between surgically and non-surgically treated individuals.

**Methods:**

We identified individuals with a lumbar disc herniation in adolescence diagnosed with magnetic resonance imaging (MRI) and contacted them for follow-up MRI. Lumbar degeneration was assessed according to Pfirrmann, Modic, and total end plate score (TEP score). Patient-reported outcome measures at follow-up comprised the Oswestry Disability Index (ODI), EQ-5D-3-level version, 36-Item Short Form Health Survey (SF-36), and Visual Analogue Scale (VAS) for back and leg pain. Fisher’s exact test, Mann–Whitney U tests, Wilcoxon tests, and logistic regression were used for statistical analysis.

**Results:**

MRIs were available at diagnosis and after a mean of 11.9 years in 17 surgically treated individuals and 14 non-surgically treated individuals. Lumbar degeneration was similar at diagnosis (P = 0.2) and at follow-up, with the exception of higher TEP scores in surgically treated individuals at levels L4–L5 and L5–S1 at follow-up (P ≤ 0.03), but this difference did not remain after adjustment for age and sex (P ≥ 0.8). There were no significant differences in patient-reported outcome measures between the groups at follow-up (all P ≥ 0.2).

**Conclusion:**

Adolescents with a lumbar disc herniation have, irrespective of treatment, a similar degree of lumbar degeneration at the time of diagnosis, and similar lumbar degeneration and patient-reported outcomes at long-term follow-up.

The incidence of surgery for lumbar disc herniation in Sweden is 29/100,000 with adolescents accounting for 1.4% of all surgeries [1,2]. The risk of additional lumbar spine surgery after surgery for lumbar disc herniation in adolescence is around 11%, and similar to the risk seen in young adults [3]. This could indicate that lumbar disc herniation in adolescence is associated with an early start of lumbar degeneration.

More than a decade after surgery, a higher degree of lumbar degeneration has been observed after lumbar disc herniation surgery in adolescence when compared with controls [4]. To our knowledge, there has been no previous research on the prevalence and progression of lumbar degeneration in adolescents (at or below the age of 18) with lumbar disc herniation. It is not known whether the degree of degeneration at the time of diagnosis in adolescents treated surgically differs from the degree of degeneration in individuals treated non-surgically. Nor is it known whether the degree of degeneration differs in the long term. The aim of this study was to compare the degree of degeneration at the time of diagnosis and in the long term for adolescents treated surgically and non-surgically for lumbar disc herniation. We hypothesized a significant increase in the degree of degeneration, irrespective of treatment.

## Methods

### Study design

The checklist STrengthening the Reporting of OBservational studies in Epidemiology (STROBE) has been used when preparing this manuscript.

Adolescents who had been treated surgically for lumbar disc herniation after a period of non-surgical management at or below 18 years of age between January 1998 and March 2011 and living in Stockholm County at the time of follow-up were identified using the national Swedish spine registry (www.swespine.se). A comparison between the identified 23 surgically treated individuals and 23 healthy controls has been reported earlier [4]. The cohort in the current study consists of 17 of the 23 individuals in whom we could identify a complete preoperative MRI taken at the time of diagnosis. All surgeries consisted of removal of the herniated disc on a single level performed with (n = 8) or without (n = 9) the use of a surgical microscope.

The non-surgically treated individuals had been diagnosed with lumbar disc herniation at or under 18 years of age between January 2004 and December 2016, and were identified based on the ICD-10 codes M51.1 and M51.1K in the hospital files of the Karolinska University Hospital, Stockholm, Sweden. All charts were screened for diagnosis verification. 63 individuals were contacted by mail and asked to participate in the study. When reviewing the group, 35 individuals were found to have had surgery for lumbar disc herniation a median 0.3 (25th–75th percentile [IQR] 0.1–1.3) years after diagnosis and were excluded.

The MRIs at diagnosis were for both groups collected from the Picture Archiving and Communications System (PACS) at Karolinska University Hospital. All disc herniations at diagnosis were retrospectively classified according to the Michigan State University (MSU) classification for size (1, 2, 3; 1 being the smallest and 3 the largest herniation) and location (a, ab, b, c; a being the most central and c the most lateral location) [5]. Signs of ring apophysis fracture were noted.

The follow-up examinations for both groups were performed at the MR Research Centre, Karolinska Institutet, Stockholm, Sweden. The follow-up MRI examinations for the 17 surgically treated individuals took place between May 2019 and January 2020, a mean 13.1 years after diagnosis. The follow-up MRI examinations for the 14 included non-surgically treated individuals took place between June 2021 and September 2021, a mean 10.5 years after diagnosis.

Follow-up imaging data was acquired with identical sequences for both groups with the use of a 3.0-T MRI scanner (Discovery MR750; GE Healthcare, Chicago, IL, USA). Imaging included sagittal T1-weighted, T2-weighted, and Short Tau Inversion Recovery (STIR) sequences of the lumbar spine. Follow-up questionnaires for both groups were retrieved in conjunction with the follow-up MRI examinations.

### Degeneration

Disc degeneration was assessed using the Pfirrmann classification on sagittal T2-weighted MRI sequences [6]. In grade 1, the disc structure is homogeneous with a normal disc height and a hyperintense white signal intensity. If the disc structure is inhomogeneous and the distinction between the nucleus pulposus and anulus fibrosus is clear, with or without horizontal grey bands, the disc is scored as grade 2. In grade 3 the disc is normal in height or slightly decreased with an inhomogeneous structure, an intermediate grey signal intensity, and the distinction between nucleus pulposus and anulus fibrosus is unclear. A disc is scored as grade 4 if the disc is inhomogeneous in its structure with a hypointense dark-grey signal intensity, lost distinction between the nucleus pulposus and anulus fibrosus, and a disc height normal to moderately decreased. Lastly, in grade 5 the disc space is collapsed, and the disc structure is inhomogeneous with a hypointense black signal intensity.

The vertebral body endplates were assessed according to Modic et al. using sagittal T1- and T2-weighted sequences and sagittal STIR sequences [7]. Type 0 represents no degenerative changes. If there is the presence of oedema, this is scored as type 1. Type 2 represents marrow replaced by fat. If there are signs of sclerosis in the subchondral plate, this is scored as type 3.

Endplate defects were assessed according to Rajasekaran et al. using sagittal T1- and T2-weighted and STIR sequences [8]. Type 1 represents no defects with a uniform hypointense band. Type 2 represents focal thinning of the endplate. In type 3, there are focal disc marrow contacts. In type 4, the defects in the endplate cover up to 25% of the endplate width. In type 5, the defects cover up to 50% of the endplate width and in type 6 there is complete endplate damage with sclerosis. Depression of disc space is usually present from type 4–6. The endplate defect types were calculated into total endplate score (TEP score) in which the defect types, above and below the disc, were summated for each segment. A TEP score of 6 or higher is associated with a high incidence of degenerative disc disease [8].

**Figure 1:**
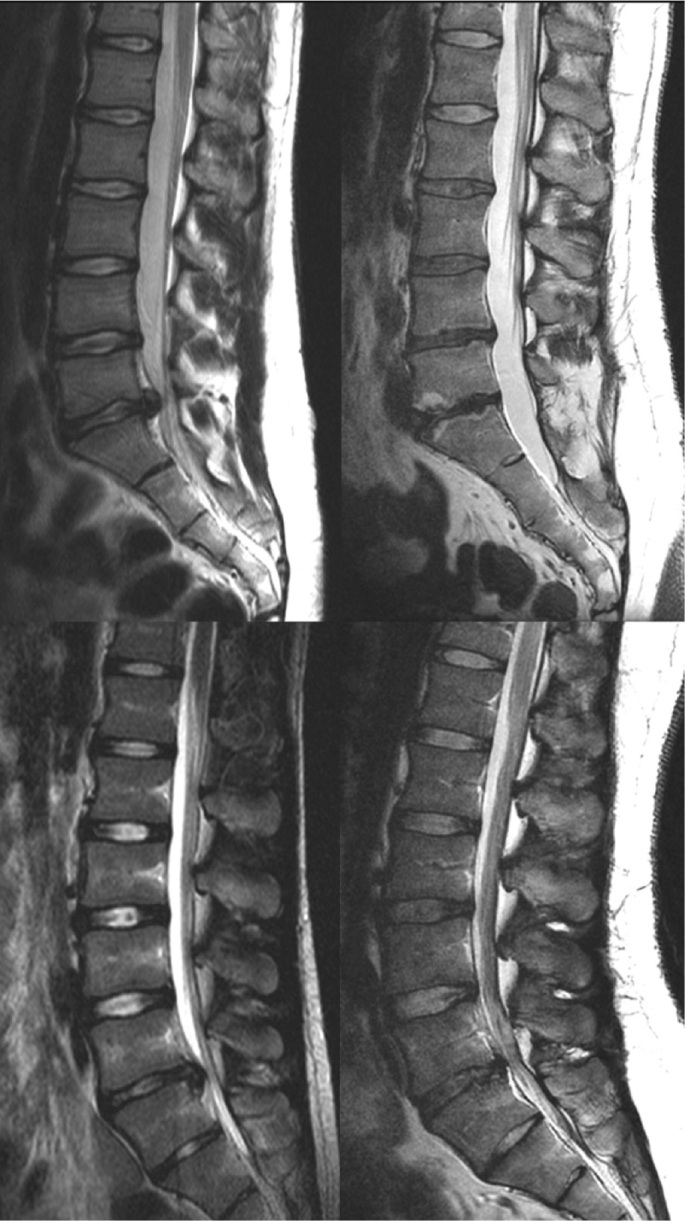
Baseline and follow-up MRI of a surgically treated individual (top) and a non-surgically treated individual (bottom) with disc herniations at level L5–S1. Baseline MRIs are shown on the left, and follow-up MRIs on the right. Degeneration according to Pfirrmann in the surgically treated individuals progressed from 1 to 3 at level L2–L3 and L3–L4, from 2 to 4 at L4–L5, and from 2 to 5 at L5–S1. Modic type 2 changes are seen at the L5–S1 level. In the non-surgically treated individuals, degeneration according to Pfirrmann progressed from 1 to 3 at L3–L4 and from 2 to 4 at L5–S1.

All MRI evaluations were blinded as to the treatment and performed by one of the authors (MS), an experienced neuroradiologist. Figure 1 show examples of MRIs.

### Patient-reported outcome measures

At follow-up, the Oswestry Disability Index (ODI) 2.1a, EQ-5D-3 level, 36-Item Short Form Health Survey (SF-36), and the Visual Analogue Scale (VAS) for back and leg pain were used.

The ODI is a measure of back-related disability and ranges between 0 (no disability) and 100 (maximum disability) (9). The ODI 2.1a version was used.

EQ-5D-3L represents the societal perspective on health and the answers are translated to an index. We used the United Kingdom time trade-off tariff (UK-TTO) ranging from −0.59 (worst) to 1.0 (best) [10].

The SF-36 is a health survey comprising 36 questions measuring the respondent’s physical and mental health. We report the summary measures Physical Component Summary Score (PCS) and Mental Component Summary Score (MCS) ranging from 0 (worst) to 100 (best) [11].

VAS for back and leg pain during the last week range between 0 (no pain) to 100 (maximum pain).

Current and previous smokers were asked to declare the number of cigarettes smoked per day and for how long they had been smoking. Pack years were calculated as the number of cigarettes per day times years smoking, divided by 20.

To assess strain related to occupation, the respondents were asked to describe their occupation by choosing one of 4 levels: (1) mainly sedentary, (2) mobile to some extent with no heavy lifting, (3) moderately heavy labor, and (4) heavy manual labor [12].

### Statistics

Data is shown as number, mean (95% confidence interval [CI]), or median with 25th to 75th percentile (IQR). For statistical comparisons of continuous independent variables and for categorical variables, the Mann–Whitney U test and Fisher’s exact test were used, respectively. The Wilcoxon signed-rank test was used for paired sample analysis of continuous variables. Logistic regression was used when adjustment for age and sex was performed. Pfirrmann grading was dichotomized into absence of degeneration (grade 1) and presence of degeneration (grade 2–5). Modic types were dichotomized into absence (type 0) or presence (type 1–3) of changes, and TEP scores were dichotomized as < 6 or ≥ 6.

Any missing data was handled by case-by-case exclusion in the analyses. Statistical analysis was performed in IBM SPSS Statistics version 28 (IBM Corp, Armonk, NY, USA) and in R, version 4.2.3 (2023-03-15 ucrt) (R Foundation for Statistical Computing, Vienna, Austria).

Significance level was set to P < 0.05.

### Ethics, registration, data sharing, funding, and disclosures

Written informed consent was obtained from all included individuals. Ethical permission for this study was given by the Ethical Review Board in Stockholm, Sweden (number 2012/206-31/1, 2018/299-31/1 and 2019-01713). PG was supported by Region Stockholm (ALF), Region Stockholm in a clinical research appointment, by CIMED, Karolinska Institutet, Uppsala University, and the Swedish Research Council. TL was supported by grants from HRH Crown Princess Lovisa’s Society for Child Care and the Swedish Society of Spinal Surgeons. SB was supported by grants from the Sven Jerring Foundation. The authors have no conflicts of interest. Complete disclosure of interest forms according to ICMJE are available on the article page, doi: 10.2340/17453674.2024.39944

## Results

**Figure 2:**
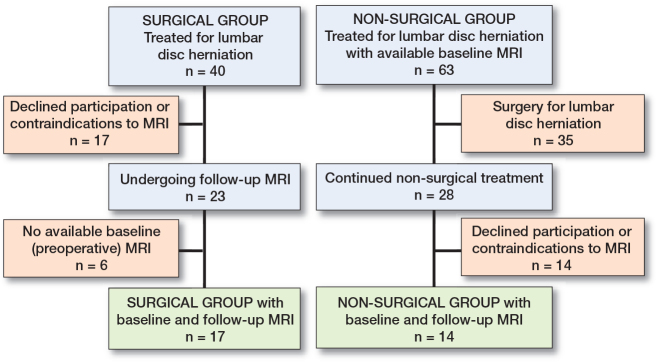
Flowchart of the participants.

We included 17 surgically treated individuals with both a baseline and a follow-up MRI, and 28 individuals with continued non-surgical treatment with a baseline MRI, of whom 14 had a follow-up MRI (Figure 2).

At baseline, surgically treated individuals were older than the non-surgically treated individuals (Table 1). There were no statistically significant differences in lumbar degeneration at baseline (Table 1), and the result was similar when only considering the levels with the largest disc herniations (data not shown, all P > 0.1). Disc herniations tended to be smaller in the non-surgical group than in the surgical group, although not statistically significant (Table 1). Ring apophyseal fractures were seen in 3 non-surgically treated individuals (Table 1).

**Table 1:** Baseline characteristics including disc degeneration according to Pfirrmann, Modic, and Rajasekaran (TEP score) in surgically and non-surgically treated individuals at baseline: size and location of the largest disc herniation is shown and based on the Michigan State University (MSU) classification (see methods for explanation) [5]. P values are given for Fisher’s exact test or Mann–Whitney U test. Values are median (IQR) unless otherwise specified

Factor	Surgically treated group (n = 17)	Non-surgically treated group Follow-up MRI		P value ^a^	P value ^b^
		yes (n = 14)	no (n = 14)		
Mean age	17.1	15.1	14.9	0.002	0.8
(CI)	16.4–17.7	13.3–16.7	14.1–17.3		
Females, n	9	10	8	0.5	0.7
Level of largest disc herniation, n					
L1–L2	0	0	1	0.1	1.0
L4–L5	5	8	7		
L5–S1	12	6	6		
Size of disc herniation, n					
1	1	2	6	0.1	0.1
2	12	10	8		
3	4	2	0		
Location of disc herniation, n					
a	4	8	8	0.09	0.5
ab	9	3	5		
b	4	3	1		
Ring apophyseal					
fracture, n	0	1	2	0.3	1.0
Pfirrmann					
L1–L2	1 (1–1)	1 (1–1)	1 (1–1)	0.6	0.8
L2–L3	1 (1–1)	1 (1–1)	1 (1–1)	1.0	1.0
L3–L4	1 (1–1)	1 (1–1)	1 (1–1)	0.7	0.8
L4–L5	2 (1–2)	2 (1–2)	2 (1–2)	0.7	1.0
L5–S1	2 (2–2)	2 (2–3)	1 (1–2.75)	0.7	0.1
Modic					
L1–L2	0 (0–0)	0 (0–0)	0 (0–0)	0.5	0.5
L2–L3	0 (0–0)	0 (0–0)	0 (0–0)	N/A	N/A
L3–L4	0 (0–0)	0 (0–0)	0 (0–0)	N/A	N/A
L4–L5	0 (0–0)	0 (0–0)	0 (0–0)	0.5	1.0
L5–S1	0 (0–0)	0 (0–0)	0 (0–0)	1.0	1.0
TEP score					
L1–L2	2 (2–2)	2 (2–2)	2 (2–2)	0.3	0.5
L2–L3	2 (2–2)	2 (2–2)	2 (2–2)	1.0	1.0
L3–L4	2 (2–2)	2 (2–2)	2 (2–2)	1.0	1.0
L4–L5	2 (2–2)	2 (2–2)	2 (2–2)	0.2	0.7
L5–S1	2 (2–2)	2 (2–2)	2 (2–2)	0.9	0.5

N/A = not applicable, tied data.

a Surgically treated group vs. all non-surgically treated.

b Non-surgically treated with MRI follow-up vs. non-surgically treated without MRI follow-up.

At the time of follow-up, the surgically treated group was aged median 30.0 (IQR 28.7–31.4) years and the non-surgically treated group was aged 25.5 (IQR 23.2–27.8) years (P = 0.002). Corresponding data for body mass index was 23.3 (IQR 21.6–24.9) and 26.1 (IQR 23.4–28.5) (P = 0.07) and for pack years 0 (IQR 0–0.3) and 0 (IQR 0–0.1) (P = 0.2). Occupational load did not differ significantly between the groups (P = 0.3). At the time of follow-up, 7 individuals in the surgical group had been operated on a second time in the lumbar spine, and 6 of these were performed at the index level. 2 of them were operated on a third time at the index level.

From baseline to follow-up, there was a statistically significant progression in degeneration within both groups according to Pfirrmann at levels L3–S1 (all P ≤ 0.04). In the surgically treated group, there was a statistically significant progression of degeneration according to Modic at level L4–L5 (P = 0.007) and TEP score at all levels (all P < 0.04).

At follow-up, there were no statistically significant differences between the groups in degeneration according to Pfirrmann or Modic, while the surgically treated individuals had a significantly higher TEP score at levels L4–S1 (Table 2). There were no significant differences in degeneration at follow-up between the groups after adjustment for age and sex in logistic regressions (data not shown, all P ≥ 0.8). The result was similar when only considering the levels with the largest disc herniations, after adjustment for age and sex (data not shown, all P > 0.1).

**Table 2:** Disc degeneration according to Pfirrmann, Modic. and Rajasekaran (TEP score) in surgically and non-surgically treated individuals at follow-up. Values are median (IQR)

Factor	Surgically treated group (n = 17)	Non-surgically treated group (n = 14)	P value ^a^
Pfirrmann			
L1–L2	1 (1–1)	1 (1–1)	0.9
L2–L3	1 (1–1)	1 (1–1)	0.9
L3–L4	1 (1–3)	1 (1–2)	1.0
L4–L5	3 (2–4)	3 (1–4)	0.5
L5–S1	3 (3–4)	3 (3–4)	0.6
Modic			
L1–L2	0 (0–0)	0 (0–0)	N/A
L2–L3	0 (0–0)	0 (0–0)	1.0
L3–L4	0 (0–0)	0 (0–0)	0.5
L4–L5	0 (0–2)	0 (0–0)	0.3
L5–S1	0 (0–2)	0 (0–0)	0.7
TEP score			
L1–L2	2 (2–4)	2 (2–2)	0.2
L2–L3	2 (2–4)	2 (2–2)	0.3
L3–L4	2 (2–4)	2 (2–2)	0.3
L4–L5	5 (2–10)	2 (2–5)	0.03
L5–S1	6 (5–10)	2 (2–5)	< 0.001

N/A = not applicable, tied data.

a Fisher’s exact test or Mann–Whitney U test.

### Patient-reported outcome measures

There were no statistically significant differences in patient-reported outcome measures between surgically treated and non-surgically treated individuals (Table 3).

**Table 3:** Comparison of VAS for back and leg pain, ODI, EQ-5D-3L, SF-36 PCS, and SF-36 MCS between surgically and non-surgically treated individuals at follow-up. Values are as mean (CI)

Score	Surgically treated group (n = 17)	Non-surgically treated group (n = 14)	P value **^a^**
VAS back	20 (9–32)	24 (12–36)	0.5
VAS leg	8 (2–14)	14 (1–27)	0.8
ODI	12 (7–17)	12 (5–19)	1.0
EQ-5D-3L	0.81 (0.71–0.91)	0.76 (0.65–0.87)	0.3
SF-36 PCS	50 (47–53)	48 (43–53)	0.6
SF-36 MCS	50 (46–54)	45 (40–51)	0.2

VAS = Visual Analogue Scale for pain, ODI = Oswestry Disability Index, SF-36 = 36-Item Short Form Health Survey, PCS = Physical Component Summary score, MCS = Mental Component Summary score.

a Mann–Whitney U-test.

### Non-responder analysis

There were no statistically significant differences in age at diagnosis or sex between the 23 responders and the 17 non-responders in the surgically treated group (P = 0.6 and P = 1.0). There were no statistically significant differences in age at diagnosis, sex, size and location of the disc herniation, or disc degeneration between the 14 responders and 14 non-responders in the non-surgically treated group (Table 1).

## Discussion

We aimed to establish whether the degree of lumbar degeneration differs at diagnosis or at follow-up between surgically and non-surgically treated individuals. We found that adolescents with a lumbar disc herniation have, irrespective of treatment, a similar degree of lumbar degeneration at the time of diagnosis, and similar lumbar degeneration and patient-reported outcomes at long-term follow-up.

The current study, and our previous studies [3,4], make it apparent that lumbar disc herniation in adolescence is indicative of an early degenerative process in the lumbar spine. Some evidence of disc degeneration was seen in the lower lumbar levels already at baseline, corresponding to the levels affected by disc herniations. That age is an important factor in the degenerative processes in the spine is well described [13-15], but the prevalence of disc degeneration in adolescents with or without lumbar disc herniation has not been completely established [16,17].

Our hypothesis of an increase in the degree of disc degeneration in both study groups with time was confirmed and expands the current knowledge base. Others have reported that the extent of disc herniation in asymptomatic individuals is related to progression of disc degeneration [18], although tear of the annulus fibrosus itself, which is likely part of the process of developing a disc herniation, is not a predictor for accelerated disc degeneration [19]. Disc degeneration is more prevalent in youths with lumbar back pain, but its association with future pain or disability is weak [17,20-23]. Longer follow-ups would help us define the relationships between disc degeneration, symptoms, and additional spine surgery.

The total endplate score was larger in the lower lumbar levels in the surgically treated group at follow-up. Endplate defects covary with disc degeneration [15,24], and a higher total endplate score is associated with progression of disc degeneration and Modic changes, and is also age-dependent [25]. The mean 4.5-year difference in age at follow-up between the 2 study groups is probably the reason for the difference, and adjustment for age resulted in non-significance in the logistic regressions. In other words, a presumably worse clinical presentation and possibly larger lumbar disc herniation in individuals admitted for surgery during adolescence was not reflected in a higher degree of degeneration at the time of presentation or at later follow-up compared with a non-surgically treated group.

### Quality of life

Several studies suggest that non-surgical treatment is less effective in adolescents than in adults [26], and our data also shows that the majority of individuals eventually underwent surgery. A proposed explanation for the less effective non-surgical management in adolescents is that the less degenerated and more viscous discs in youth take longer to resorb [27]. Even though a large number of adolescent patients with lumbar disc herniations are eventually treated with surgery, the treatment is considered successful [4,28,29]. Nonetheless, even though most of the patients are satisfied with their surgery, they experience more back pain, disability, and lower quality of life compared with healthy controls [4].

At follow-up, there were no differences in quality-of-life measures between the surgically and the non-surgically treated groups. The finding that patient-reported outcome in surgically and non-surgically treated individuals is similar in the long-term is consistent with studies on adults [30,31]. This strengthens the evidence of non-surgical treatment as an effective treatment for lumbar disc herniation in adolescents also, notwithstanding the possible differences of outcome in the short term. In addition, non-surgical treatment is not associated with the risk of complications that surgery poses, making it the first-line treatment [26].

### Strengths and limitations

The strengths of this study are the long follow-up time, and the use of validated and well-established outcome measures.

Limitations are as follows. The sample sizes were small, as the response rates were low from a relatively small group of individuals with a rare condition. The non-responder analysis indicates that at least there were no sex, disc herniation, or disc degeneration related differences at the time of diagnosis. Individuals were included based on age and not maturity status, so any associations between maturity and disc degeneration at baseline cannot be determined. Cross-sectional data from the Swespine registry covers only surgically treated individuals. Thus, comparison of patient-reported outcome measures at baseline between the groups is not feasible. It is likely that the individuals who underwent surgery had more symptoms than the non-surgically treated individuals.

### Conclusions

Our study showed that the degree of lumbar degeneration is not associated with the choice of treatment. Furthermore, as seen in adults, surgically and non-surgically treated individuals report similar patient-reported outcome measures in the long term. Surgery does not seem to accelerate lumbar degeneration compared with non-surgical treatment.

## Notes

TL, MS, KJ and PG conceived the idea, designed and directed the study and are accountable for all aspects of the work. SP and TL collected questionnaire and patient data. SP did the statistical analyses and drafted the manuscript with input from TL and PG. SP, SB, and TL performed the MRI examinations, supervised by TL and KJ. MS analyzed all MRI examinations. All contributed with interpretation of the results and manuscript comments. PG edited the manuscript before submission and is the corresponding author. PG supported the study with grants for the ethical application, data retrieval and external statistical advice.

Handling co-editor: Ilkka Helenius

*Acta* thanks Matti Ahonen, Muharrem Yazici, and one anonymous reviewer for help with peer review of this manuscript.
